# In this issue of *Current Oncology*

**Published:** 2008-10

**Authors:** R.J. Ablin

In addition to a line-up of topical articles, we are delighted that our newest section, Drug Development in Contemporary Oncology, under the editorship of Michel Tremblay (Goodman Cancer Centre of McGill University), makes its debut in this issue of *Current Oncology.* The first of a forthcoming series of mini-reviews covers the histone deacetylase inhibitors. Donald Walkinshaw and Xiang-Jiao Yang from the Goodman Cancer Centre of McGill University present a superb overview of the basic and clinical aspects of this exciting new class of compounds for the treatment of cancer.

In the section Oncologic History, Donald Cowan (Cancer Care Ontario and Sunnybrook Health Sciences Centre) provides an account of the invaluable pioneering contributions by Vera Peters to the diagnostic staging and treatment of Hodgkin disease. In the course thereof, Dr. Cowan further provides the historical backdrop to Peters’s work, featuring some of the other prominent researchers who contributed to improvements in the treatment of Hodgkin disease. Ironically, Dr. Peters died of metastatic cancer in Princess Margaret Hospital, where she served for many years.

Bone metastasis, the third most frequent complication of cancer (closely trailing liver and lung metastasis), but oftentimes first as related to the primary cancer, is the subject of two articles in this issue. In a comparative analysis (with literature review) of patients receiving palliative radiotherapy for bone metastasis, Amanda Hird and colleagues (Odette Cancer Centre) observed similar rates of symptomatic pain relief in patients with gastrointestinal (gi) primary cancer as in those with metastasis from other primary cancer sites. Based on their observations, Hird *et al.* emphasize that “patients with symptomatic bone metastases from gi malignancies should be referred for palliative radiotherapy as readily as patients with osseous metastases from other primary cancer sites.” In the second report, Stephanie Hadi and colleagues (also of the Odette Cancer Centre) aimed to validate “symptom clusters” (scs) among the factors interfering with physical and psychological functions in patients receiving palliative radiotherapy for their symptomatic bone pain. These authors found that the scs seen in recent patients were not the same as those seen in a previous study group. Before making any final conclusions on the usefulness of scs, the authors suggest that evaluation in a larger patient cohort is necessary.

Alan Nyitray (University of Arizona College of Public Health) contributes to the Updates and Developments in Oncology section with a brief but concise overview of human papillomavirus (hpv) in heterosexual men. Vaccination for hpv will be the subject of an upcoming supplement to the journal.

Wilson Cheung *et al.* (University of Toronto and Odette Cancer Centre) bring us a unique caveat with regard to ammonia levels in neuropsychiatric complications of 5-fluorouracil (5-fu) therapy for colon cancer. Their case report is additionally unique in describing the first patient to develop 5-fu encephalopathy as a result of folfox chemotherapy, which has recently become the new standard of care in therapy for colorectal cancer.

Patients with chronic lymphocytic leukemia (cll) are at increased risk for secondary malignancies, for which the mortality rate is high. Among these secondary malignancies, squamous cell carcinoma (scc) is particularly problematic. In a report of four cases of scc secondary to cll, Jennifer Wong and colleagues (Odette Cancer Centre) present findings of a favourable indication for radiotherapy after local recurrence following surgical excision.

Our recently instituted method of publishing in a combination of hard copy and electronic means has permitted us to bring you three additional interesting articles in this issue. To begin, a second report by Jennifer Wong and colleagues (Odette Cancer Centre) provides a comprehensive literature review of quality of life (qol) results from trials of whole-brain radiotherapy used alone and in combination with other treatments for brain metastasis. The authors emphasize the importance of including qol as an endpoint in future trials, so that a better understanding can be obtained concerning the role of qol, particularly for improving treatment of patients with brain metastasis. In a second online article, Max Dahele and colleagues (Princess Margaret Hospital, University of Toronto, and Sunnybrook Health Sciences Centre) meticulously report, using color images, their initial experience with an innovative approach for three-dimensional correlation of positron-emission and computed tomography images with whole-mount histopathology in non-small-cell lung cancer. Their methodologic principles may be readily transferable to other tumour types. Finally, Flynn *et al.* (Sunnybrook Health Sciences Centre) present two cases of acrometastasis to the hands, and an extensive review of the literature. With lung cancer being the number one primary cancer site metastasizing to the hands (followed by kidney and breast cancers), the well-documented report by these authors confirms that a diagnosis of hand metastasis indicates poor prognosis, average survival being 6 months.

We at *Current Oncology* continue to strive to bring you timely and topical articles. However, we need to hear your comments on whether we are succeeding in that endeavour and your suggestions of specific topics that you would like to see covered in the journal. We welcome your comments, which should be sent to Laura Shand at current_oncology@multi-med.com.

